# Characterization of Binding Properties of Individual Functional Sites of Human Complement Factor H

**DOI:** 10.3389/fimmu.2020.01728

**Published:** 2020-08-04

**Authors:** Aftabul Haque, Claudio Cortes, M. Nurul Alam, Maladi Sreedhar, Viviana P. Ferreira, Michael K. Pangburn

**Affiliations:** ^1^Center for Biomedical Research, University of Texas Health Science Center, Tyler, TX, United States; ^2^The Picower Institute for Learning and Memory, Massachusetts Institute of Technology, Cambridge, MA, United States; ^3^Department of Foundational Medical Sciences, Oakland University William Beaumont School of Medicine, Rochester, MI, United States; ^4^Department of Biology, College of Arts, Sciences, and Education, Texas A&M University-Texarkana, Texarkana, TX, United States; ^5^Department of Medical Microbiology and Immunology, University of Toledo College of Medicine, Toledo, OH, United States

**Keywords:** complement, Complement Factor H, immunology, innate immunity, protein expression, structure-function

## Abstract

Factor H exists as a 155,000 dalton, extended protein composed of twenty small domains which is flexible enough that it folds back on itself. Factor H regulates complement activation through its interactions with C3b and polyanions. Three binding sites for C3b and multiple polyanion binding sites have been identified on Factor H. In intact Factor H these sites appear to act synergistically making their individual contributions difficult to distinguish. Recombinantly expressed fragments of human Factor H were examined using surface plasmon resonance (SPR) for interactions with C3, C3b, iC3b, C3c, and C3d. Eleven recombinant proteins of lengths from one to twenty domains were used to show that the three C3b-binding sites exhibit 100-fold different affinities for C3b. The N-terminal site [complement control protein (CCP) domains 1-6] bound C3b with a *K*_*d*_ of 0.08 μM and this interaction was not influenced by the presence or absence of domains 7 and 8. Full length Factor H similarly exhibited a *K*_*d*_ for C3b of 0.1 μM. Unexpectedly, the N-terminal site (CCP 1-6) bound native C3 with a *K*_*d*_ of 0.4 μM. The C-terminal domains (CCP 19-20) exhibited a *K*_*d*_ of 1.7 μM for C3b. We localized a weak third C3b binding site in the CCP 13-15 region with a *K*_*d*_ estimated to be ~15 μM. The C-terminal site (CCP 19-20) bound C3b, iC3b, and C3d equally well with a *K*_*d*_ of 1 to 2 μM. In order to identify and compare regions of Factor H that interact with polyanions a family of 18 overlapping three domain recombinant proteins spanning the entire length of Factor H were expressed and purified. Immobilized heparin was used as a model polyanion and SPR confirmed the presence of heparin binding sites in CCP 6-8 (*K*_*d*_ 1.2 μM) and in CCP 19-20 (4.9 μM) and suggested the existence of a weak third polyanion binding site in the center of Factor H (CCP 11-13). Our results unveil the relative contributions of different regions of Factor H to its regulation of complement, and may contribute to the understanding of how defects in certain Factor H domains lead to disease.

## Introduction

Complement Factor H plays an essential role in homeostasis of the complement system. It controls the spontaneous fluid phase activation of the complement alternative pathway and it prevents activation of this pathway on host surfaces (1) through specific interactions with host markers (1–6). In addition, many human pathogens use receptors to bind Factor H thus minimizing complement activation and enhancing their survival (1, 7). Control of complement activation on host tissues involves interactions of Factor H with surface-bound C3b, iC3b, C3d, with polyanionic host markers (4, 6, 8) and with malondialdehyde (MDA)-modified lipids or proteins (9). Interactions of polyanions with one or more of the polyanion binding sites on Factor H appears to be the primary mechanism of recognition of host cells and tissues (1, 8).

Mutations in Factor H as well as allotypes common in the human population have been linked to human diseases. Age-related macular degeneration (ARMD) has been strongly linked to a single amino acid allotype in domain 7 (Y402H in CCP 7) (10). This region has been shown to bind polyanions, CRP, and MDA and this allele affects these interactions (1). Approximately 35% of the human population carries at least one copy of this allele. A less prevalent, but often fatal group of mutations affecting CCP 19-20 have been linked to inherited hemolytic uremic syndrome (aHUS) (11, 12). The CCP 19-20 region of Factor H contains binding sites for C3b, iC3b, C3d, MDA, and for polyanionic markers prevalent on human cells and tissues (4, 5, 8, 13–15). Additional binding sites for C3b and polyanions have been reported in the center of the protein (16–19), but the evidence for these has not been consistent. Evidence is presented here localizing both of these binding sites.

The primary function of Factor H in the complement system is to bind to C3b and control progression of the complement cascade. All three pathways of complement activation require activation of native C3 to form C3b and all three pathways utilize C3b to activate C5. Activation of C5 releases the anaphylatoxin C5a and initiates formation of the membrane attack complex of complement. Regulation of C3b is critical to homeostasis and the mutations in the Factor H gene associated with aHUS and ARMD appear to result in activation of complement due to the changes in tissue specific host recognition sites on Factor H and FHL-1 (1, 4, 5, 8, 12, 14, 20). Microorganisms typically lack such markers and thus bind Factor H poorly. As a result, spontaneously deposited C3b is amplified forming focal points of complement activation. This amplification process of the alternative pathway is capable of opsonizing bacteria, yeast, or parasites with millions of covalently attached C3b molecules within minutes of the microorganism's initial contact with blood (21).

Factor H inactivates C3b and the complement cascade by utilizing two functions both found in the N-terminal CCP domains (1, 22–25). If Factor H binds to C3b before the assembly of other complement proteins (Factors B and P) on C3b, it both prevents subsequent assembly of activating complexes and makes the C3b susceptible to permanent inactivation by Factor I, a serine protease. If Factor H binds after assembly of active C3- and C5-cleaving enzyme complexes, it promotes the rapid dissociation of the catalytic subunit, Bb, and then promotes permanent inactivation of C3b by Factor I, producing iC3b. Additional proteolytic cleavage of iC3b releases the soluble C3c subunit leaving C3d covalently bound to the cell.

Factor H possesses multiple binding sites for the ligands C3b, iC3b, and C3d and functionally distinct binding sites for polyanions (14–17, 26–30). This paper describes a comparative analysis of the relative affinities (K_d_) of each of these sites for their ligands. It is thought that the interactions at multiple sites act synergistically to influence the regulatory effectiveness of Factor H. Using a variety of recombinant proteins of various lengths, we examined the influence of neighboring domains on affinity including the effect of the macular degeneration-related polyanion-binding site in CCP 7 site on the affinity for C3b at the N-terminal. The relative affinity of the three C3b binding sites is compared. Finally, we have utilized a family of overlapping, three-domain, recombinant proteins spanning the entire length of Factor H to localize and compare the relative affinity of its three polyanion binding sites for heparin.

## Materials and Methods

### Reagents

Complement protein Factor H was purified from normal human plasma as previously described (31). Purified Factor H was >97% homogenous by polyacrylamide gel electrophoresis with an apparent molecular weight on SDS gel electrophoresis of 155,000 in its reduced form. Protein concentration was determined spectrophotometrically using an E_280nm_ (1% solution) of 12.4 for Factor H and its recombinant fragments. Phosphate-buffered saline (PBS) was 10 mM phosphate, 145 mM NaCl, 0.02% sodium azide and pH 7.4. Veronal-buffered saline (VBS) was 5 mM veronal, 145 mM NaCl, 0.02% sodium azide, and pH 7.4. Gelatin VBS (GVB) was VBS containing 0.1% gelatin, while GVBE was GVB containing 10 mM EDTA. HEPES-buffered saline (HBS-P) contained 10 mM HEPES, pH 7.4, 150 mM NaCl, 0.005% P20 surfactant (GE Biosciences), and 0.02% sodium azide. All buffers were made to be similar and close to physiological isoelectric strength and the physiological pH to allow comparisons between measurements to be made.

### Preparation of C3 and C3-Derived Fragments

C3 was purified from normal human plasma as previously described (32). Complement component C3 which had been stored frozen at −75°C was repurified on a Mono S column (GE Biosciences) immediately prior to use to eliminate C3b-like C3 that is formed during freezing and thawing (33). C3b was prepared from fresh C3 by incubating it with Factors B and D in the presence of NiCl_2_. (100 mg C3 (5–10 mg/mL in VBS), 1 mg Factor B, 10 μg Factor D, 0.15 mM NiCl_2_ were incubated 60 min at 37^o^C and purified by gel filtration over BioGel 0.5 m Agarose. Traces of Factor B were removed by passage over anti-Factor B-Agarose). The C3b produced was converted to iC3b by incubation with Factors H and I which cleave and inactivate C3b to form iC3b (100 mg C3b (5–10 mg/mL) was incubated with 1.6 mg Factor H and 0.6 mg Factor I for 60 min at 37^o^C and purified by gel filtration over BioGel A0.5m. Traces of FH and FI were removed by passage over anti-Factor H-Agarose and anti-Factor I-Agarose). iC3b was cleaved into two fragments, C3c and C3d, by incubation with 0.1% trypsin (100 mg iC3b at 4 mg/mL was incubated with 25 μg trypsin for 15 min at 37^o^C after which 50 μg of Soybean trypsin inhibitor was added and the C3c and C3d fragments separated by gel filtration over Biogel A0.5 m. Traces of SBTI and trypsin were removed by anion exchange chromatography over Mono Q). The protein concentration of C3b and each fragment was determined spectrophotometrically using an E_280nm_ (1% solution) of 11.0. All purified proteins were stored at −75°C. M_r_ values employed in the calculations were 185,000 for C3, 176,000 for C3b, 176,000 for iC3b, 138,000 for C3c, and 34,000 for C3d.

### Preparation of Recombinant Factor H and rH Fragments

**Figure 1:**
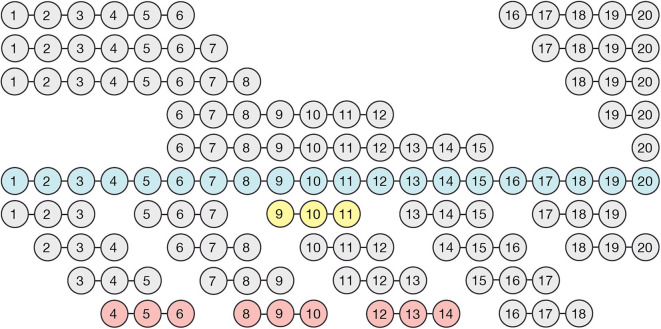
Cloning and expression of fragments spanning the full-length of Factor H. The illustrated recombinant proteins were generated using primers that begin and end in the inter-domain spacers and an expression vector designed specifically to express CCP domains as briefly described in Materials and methods. Full length Factor H (blue), and all of the Factor H sub-fragments depicted above it, were expressed in insect cells, whereas the three-domain fragments depicted below full length Factor H were expressed in the yeast system. Three constructs (shown in red) did not express in the yeast (Pichia) system and one expressed poorly (yellow). Nevertheless, as shown, the entire length was covered so that each domain was expressed in at least twice in the resulting family of proteins.

Recombinant Factor H (rH) fragments bearing a C-terminal 6His-tag were prepared as previously described (34) using a modification of the pIB/V5-His-TOPO vector from Invitrogen (Life Technologies). Full-length recombinant Factor H and rH 16-20, rH 17-20, rH 18-20, and rH 19-20 bearing a C-terminal 6×His-tag were expressed similarly in insect cells (Figure 1). Specific regions of human Factor H cDNA (35) were amplified by PCR and the inserts were cloned into the modified vector at the Sap I sites (34). The vectors were cloned in TOP 10 bacteria (Invitrogen), the fidelity of each plasmid was verified by sequencing, and the plasmid DNA was transfected into High Five insect cells according to the manufacturer's directions (Invitrogen). Stable transformants were selected with blasticidin for 6–9 days in serum free medium. Expression of proteins was done in shaker cultures at 27°C in serum free media (Sf-900 II SFM (Invitrogen) under constant 10 μg/mL Blasticidin selection. Supernatants were collected by centrifugation and either applied to and eluted from SP-Sepharose FF (GE Biosciences) as a first capture step or applied directly to Ni-loaded NTA-agarose (Qiagen). Proteins bound to Ni-NTA-agarose were eluted with an imidazole gradient from 0–150 mM imidazole at pH 8.0. Recombinant protein-containing fractions were identified using an ELISA assay employing capture on Ni-NTA microtiter plates (Qiagen) and detection with anti-V5 HRP-coupled antibody (Invitrogen). The fractions containing the recombinant proteins were pooled into 5,000 or 10,000 MWCO Amicon Ultra (Millipore) ultrafilters and concentrated by centrifugation. Proteins were dialyzed into PBS and stored frozen at −75°C until used. Yields were generally low at approximately 500 μg protein from 5 L of culture media.

Recombinant Factor H fragments containing three CCP domains and spanning the entire length of Factor H (Figure 1) were expressed in Pichia pastoris using the pPICZα vector (Life Technologies). Briefly, the original multiple cloning site of pPICZ-α A was replaced by inserting a double stranded oligo containing a SapI site flanked by overhang 5′ XhoI and 3′ XbaI sites into the corresponding Xho and XbaI sites located in the vector, as described in detail previously (34, 36) generating pPICZ-α A SAPI vector. The coding sequence for residues described in Figure 1 (bottom section) were PCR amplified from a full-length FH cDNA template using primers containing SAPI restriction sites as previously described (37). The *SapI*-digested PCR products were cloned into a pPICZ-α SAPI vector (Invitrogen).

The vectors with 3 CCP domain inserts were cloned in TOP 10 bacteria (Invitrogen), the fidelity of each plasmid was verified by sequencing, and the plasmid DNA was electroporated into yeast. Expression was directed to the secretory pathway using the yeast α-factor secretion sequence. Recombinant proteins were purified from culture supernatant using Ni-NTA-agarose or cation exchange chromatography on Source 30S (GE Healthcare) at pH 6.0. All purified proteins were stored at −75°C in PBS. Many of the expressed proteins were heterogeneous on SDS gels due to extensive glycosylation by yeast. These proteins were treated with Endo H (BioLabs) and re-purified. Protein concentrations were determined by A280 using the calculated extinction coefficient for each 3 CCP domain protein based on its amino acid sequence.

### Preparation and Use of Protein-Coated Chips for Surface Plasmon Resonance (SPR)

Factor H fragments bearing C-terminal 6His-tags were attached to Ni-charged NTA sensor chips and analyzed using a BIAcore X or a BIAcore 3,000 instrument (GE Healthcare). The Ni-NTA chips were loaded with 20 μL of His-tagged rH fragment at 3 μg/mL in HBS-P buffer at a flow rate of 5 μL/min. Approximately 500 to 1,000 RU of rH fragment was loaded onto flow cell 1 and it was determined that the binding of the 6His rH 19-20 fragment to Ni-NTA was stable during the 10 min binding experiment (i.e., <2% loss of bound ligand/10 min at 25°C). Binding of rH 1-6 was less stable (~5% loss/5 min), but corrections were made for this loss. Flow cell 2 was loaded with nickel only. After loading of the rH fragment a new stable baseline was established and binding of the C3-derived protein being tested (also in HBS-P buffer) was injected over both flow cells. After the equilibrium level of binding was reached with each concentration of C3 protein, the chip was washed with HBS-P and cleaned with 300 mM imidazole. The Ni-NTA chip was reloaded with same amount of 6His rH fragment for each subsequent injection of different concentrations of C3 fragments.

### Preparation and Use of Heparin-Coated Chips for SPR

Commercial heparin (Sigma-Aldrich porcine heparin number H3393) was modified as previously described (38) to possess a single free amino group at the reducing end while retaining all of its charged sulfate groups. This heparin was coupled via the single free amino group to flow cell 1 of a CM5 BIAcore chip using the manufacturer's procedure for amine coupling (GE Healthcare). Flow cell 2 was mock derivatized and blocked with ethanolamine. Binding interactions were determined by passing protein samples simultaneously over both the mock-derivatized flow cell and the flow cell with immobilized heparin so as to obtain the response units for binding after subtraction of the background. Samples of 20 μL of rH fragments were loaded at 3 μM in HBS-P buffer at a flow rate of 5 μL/min at 25°C.

### Analysis of Binding Data

Dissociation constants (K_d_) were determined from the analysis of equilibrium binding measured as a function of the concentration of the analyte. The binding data were analyzed according to a one-site binding equation ([Bound] = (Capacity)^*^[Free]/(K_d_ + [Free]) using non-linear regression analysis, and the binding constant (K_d_) and standard errors were determined using Grafit version 5.0 software (Erithacus Software, London, UK). The binding curves were aligned using BIA Evaluation version 3.0 software (GE Healthcare).

## Results

### Cloning, Expression and Purification of Factor H Fragments

**Figure 2:**
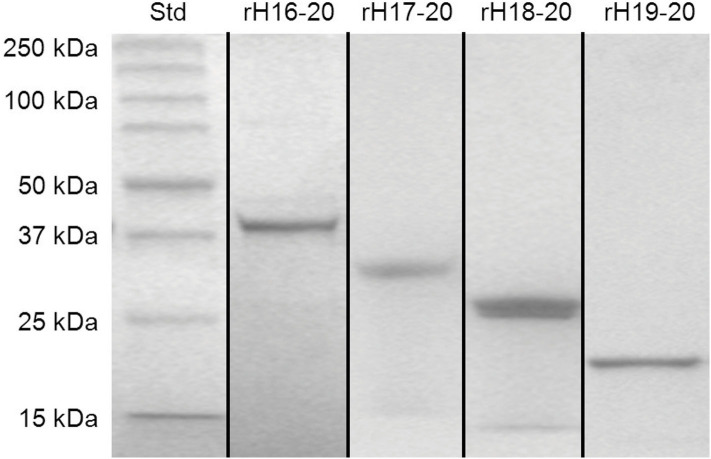
SDS-PAGE of purified fragments of human Factor H. Recombinant proteins were expressed in High Five insect cells grown in serum free media. The proteins were purified from the media by ion exchange and affinity chromatography. Proteins spanning the C-terminal domains of Factor H (rH 16-20, rH 17-20, rH 18-20, and rH 19-20 with calculated molecular weights of 39, 32, 26, and 19 kDa, respectively), and standards with the indicated size were electrophoresed through 8–16% gradient polyacrylamide gels under reducing condition and stained with Coomassie blue dye. Figure is a composite of multiple gels.

Recombinant Factor H domains expressed in High Five insect cells were grown in serum-free media and purified in two steps. Because of substances in spent serum free media that interfere with Ni-NTA affinity chromatography, the expressed proteins were first adsorbed to SP-Sepharose at low ionic strength, eluted with high salt and subsequently subjected to affinity chromatography on Ni-NTA-agarose columns. Figure 2 shows the results of cloned, expressed, and purified C-terminal fragments of Factor H (rH 16-20, rH 17-20, rH 18-20, and rH 19-20) on 8–16% gradient SDS-PAGE gels. The purified proteins ran at the expected size and contained a C-terminal V5 epitope followed by a 6X His tag. Other fragments used in this study (rH 1-6, rH 1-7, rH 1-8, rH 6-12, and rH 6-15) were expressed and purified similarly. Domain integrity was maintained by designing the PCR primers in a way that the first and last domains in the multi-domain proteins terminated in the inter-domain linker between domains as previously described (34).

### Measurement of the K_d_ of C3b for Different Factor H Sites

**Figure 3:**
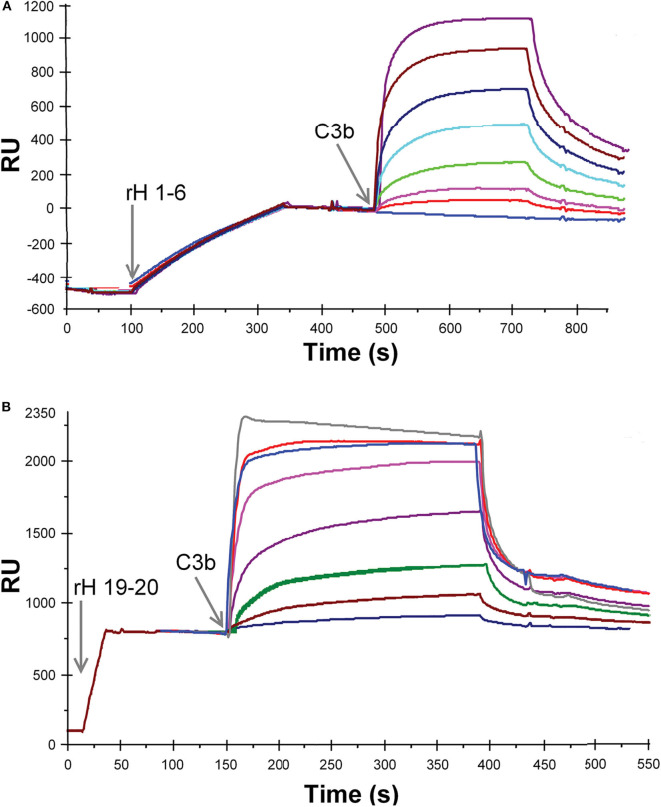
SPR analysis of the binding of recombinant Factor H to C3b. **(A)** Purified rH 1-6 was attached to the NTA-Ni-coated sensor surface by injecting 20 μl of protein (1.2 μg/ml, in HBS-P) at a flow rate of 5 μl/min over flow cell 1. Eight different concentrations of C3b (0, 0.014, 0.028, 0.055, 0.11, 0.22, 0.44, and 0.88 μM C3b) in HBS-P were injected at 5 μl/min over flow cells 1 and 2. Lower concentrations of the ligand (C3b) were used than in **(B)** due to the higher affinity between rH 1-6 and C3b. **(B)** Same as in **(A)** except that purified rH 19-20 was attached to the NTA-Ni surface by injecting 20 μl of protein (3 μg/ml, in HBS-P) at a flow rate of 20 μl/min over flow cell 1. Eight different concentrations of C3b (0.14, 0.28, 0.57, 1.1, 12.0, 2.8, 4.3, 5.7 μM C3b) in HBS-P were injected at 5 μl/min over flow cells 1 and 2 (reference cell with Ni-NTA but no rH 19-20). RU (response units) shown represent the signal from flow cell 1 minus that from flow cell 2. The overlay plot was constructed using BIA Evaluation software. After each concentration of C3b was injected, the flow cells were regenerated with 300 mM imidazole.

**Figure 4:**
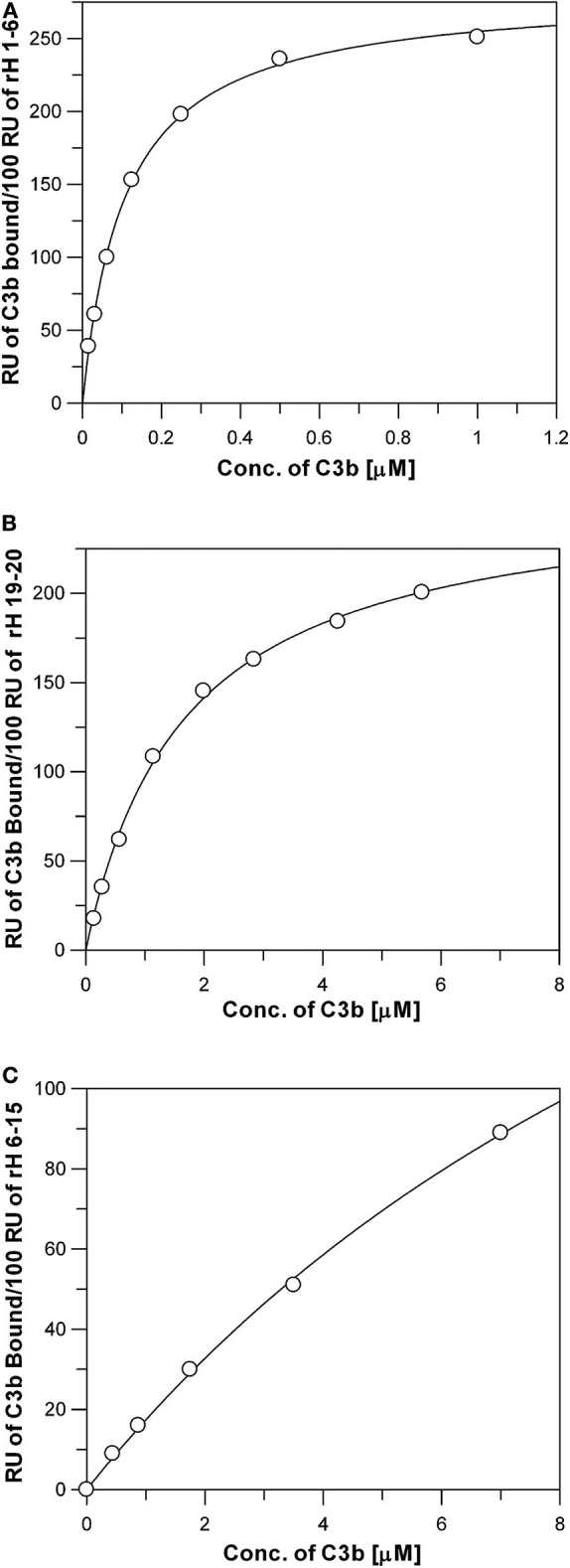
Measurement of the affinity between C3b and the three C3b binding sites of Factor H. Each data point represents the equilibrium saturation level from one individual ligand binding curve from experiments like those described in Figure 3. **(A)** rH 1-6 bound to the Ni-NTA chip exhibited the highest affinity and required C3b concentrations from 0.014 to 0.88 μM to approach saturation. The *K*_*d*_ was determined by fitting the data using non-linear regression to a single site ligand binding equation (Bound = (Capacity^*^[Free])/(*K*_*d*_ + [Free])) using GraFit 5 program (*Erithacus*). C3b was dialyzed into HBS-P. **(B)** Same as **(A)**, except purified rH 19-20 was attached to the Ni-NTA surface, **(C)** Same as **(A)**, except purified rH 6-15 was attached to the Ni-NTA surface. Higher concentrations of C3b were required in **(B,C)** due to the lower affinity of these proteins for C3b.

Ten different recombinant Factor H protein fragments were analyzed by SPR for their affinity for C3b. The method used to measure these interactions relied on the attachment of the recombinant Factor H fragments to the Ni-NTA-coated surface of BIAcore sensor chips. The purified proteins, rH 19-20 or rH 1-6, were injected over the Ni-NTA-coated surface of flow cell 1 on a BIAcore 3,000 instrument at a concentration of 3 μg/mL in 20 μL of HBS-P. Adsorption levels were highly reproducible as shown on the left sides of Figures 3A,B. The resulting Ni-NTA-6His complexes were stable during washing with buffer. In the case of rH 1-6 (Figure 3A) less that 5% of the protein decayed over the 5 min binding assays, while with rH 19-20 (Figure 3B) ~2% of the bound protein decayed during the 5 min assay. In each case corrections were made for non-specific binding by subtracting binding to the control flow cell to which no rH fragment was attached. After each different concentration of C3b was injected, the sensor chip was washed with 300 mM imidazole in HBS-P buffer to remove protein. Before the next C3b sample was injected the sensor chip was re-loaded with the rH fragment. C3b concentrations from 0.014 to 0.88 μM were injected over chips loaded with rH 1-6 to obtain data for plotting a saturation curve (Figures 3A, 4A). A similar experimental approach was used to measure the affinity of the C3b binding site at the C-terminal of Factor H (rH 19-20, Figures 3B, 4B). As evident from Figure 3A binding of the rH 1-6 fragment to the Ni-NTA-coated sensor surface was not as tight as that of rH 19-20. Corrections for the decay of rH 1-6 (~5% of maximum binding/5 min) were made to determine the degree of saturation by C3b. Due to the lower affinity of C3b for rH 19-20, C3b concentrations from 0.14 to 5.7 μM were used (Figure 3B) to determine the saturation binding curve (Figure 4B).

The binding data was generated for all the recombinant Factor H fragments from experiments similar to those shown in Figure 3. The binding data was fit as shown in Figure 4 by non-linear regression analysis to a one-site binding equation ([Bound] = (Capacity)^*^[Free]/(K_d_ + [Free]) using GraFit version 5.0 software (Erithacus Software). Table 1 lists the affinities for C3b measured for ten recombinant proteins. Factor H has been reported to possess three binding sites for C3b (17, 19, 39). The locations of two of these are well-established, however, the existence of the third site is controversial. The data in Table 1 presents the relative affinity differences between these sites. In the case of the N-terminal site the three K_d_ values measured show the lack of influence of neighboring polyanion site located in domain 7 on the affinity of the binding site in domains 1-6 due to the fact that the K_d_ is nearly identical for rH 1-6, rH 1-7, and rH 1-8 (the cDNA used in this study contained the Y402 variant). Similarly, the presence of CCP domains 16, 17, or 18 next to domains 19 and 20 makes little or no significant difference to the measured K_d_ of the C-terminal site located in domains 19-20. No detectable affinity was found for domain 20 alone. Evidence supporting the existence of a C3b site in the center of Factor H was found, but the affinity between C3b and rH 6-15 was low and could only be estimated at 15 μM (Figure 4C and Table 1). Thus, the interaction of this site with C3b is ~100-fold weaker than the affinity of the N-terminal site, but only 6- to 9-fold weaker than the interaction at the C-terminal CCP 19-20 site. The C-terminal rH 19-20 site exhibits an affinity for C3b ~10- to 20-fold weaker than the N-terminal site and yet functional studies (39–41) and disease correlations with atypical HUS (12) make it clear that the biological effects of this site have a major impact on the control of complement and the *in vivo* activation of the complement system (8). The low affinity of the central C3b binding site explains why other reports have discounted observations of weak binding in this region or reported no binding (19, 41). Nevertheless, with a Factor H serum concentration of approximately 3 μM and the potential of cooperative binding with the other two C3b binding sites, the function of Factor H may be significantly influenced by this central site as suggested by numerous studies (17, 39, 40). This is the first quantitative report of the relative affinity of all three C3b binding sites of Factor H and these measurements should aid in understanding the complex biological and disease-related functions of this protein.

**Table 1:** Binding^a^ of C3b by different regions of Factor H.

**Factor H domains**	***K_***d***_* (μM)^**b**^**	**Factor H domains**	***K_***d***_* (μM)^**b**^**
CCP 1-6	0.08 ± 0.04	CCP 16-20	2.4 ± 0.3
CCP 1-7	0.08 ± 0.02	CCP 17-20	2.1 ± 0.2
CCP 1-8	0.15 ± 0.01	CCP 18-20	1.9 ± 0.3
CCP 6-15	15 ± 2.0	CCP 19-20	1.7 ± 0.3
CCP 6-12	NDB^c^	CCP20	NDB^c^

a *Binding data was generated as in Figure 3 and K_d_ values were determined as in Figure 4 for the recombinantly generated Factor H fragments indicated*.

b *Standard errors from non-linear regression analysis are reported*.

c *NDB indicates “No Detectable Binding” was observed*.

### Measurement of the K_d_ of Full-Length Factor H for Soluble C3b

**Figure 5:**
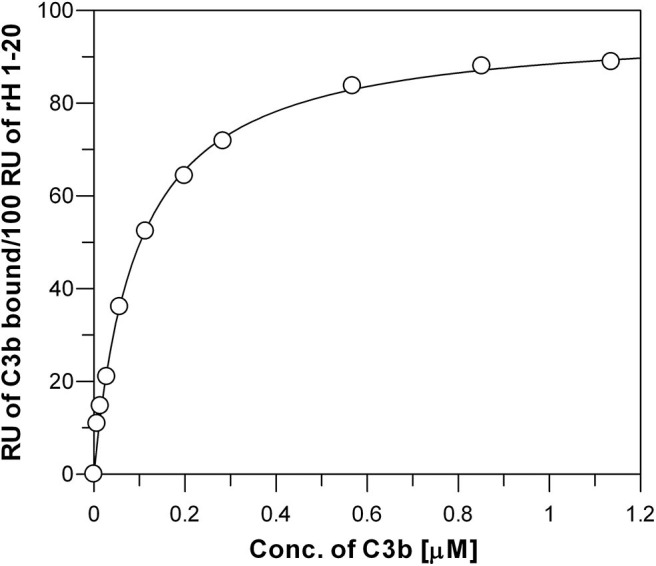
Measurement of the affinity between C3b and surface-bound full-length Factor H. Each data point represents the equilibrium saturation level from one individual ligand binding curve from experiments like those described in Figure 3. Recombinantly expressed rH 1-20-6xHis was bound to Ni-NTA BIAcore chip and different concentrations of C3b in HBS-P were injected over the chip. The *K*_*d*_ (0.095 + 0.005 μM) was determined by fitting the data using non-linear regression to a single site ligand binding equation (Bound = (Capacity^*^[Free])/(*K*_*d*_ +[Free])) using GraFit 5 program (*Erithacus*).

The affinity of the three individual sites in Factor H for C3b was measured (Figures 3, 4) by binding the CCP domains to a Ni-NTA-coated chip. Full-length Factor H expressed in insect cells with a 6×His tag at the C-terminal end was similarly coupled and the affinity was determined as shown in Figure 5 to be 0.095 + 0.005 μM. This is nearly identical to the affinity measured for the highest affinity binding site rH 1-6 located at the N-terminal end of Factor H (Figure 4A and Table 1). Because C3b was the free ligand, multiple site binding was not possible as it would be with multiple C3b molecules on a surface and free Factor H. Thus, one would expect the highest affinity site to dominate the measurement in this arrangement and both rH 1-6 and rH 1-20 exhibited a K_d_ of ~0.1 μM. This assumes that Factor H cannot use multiple sites and “wrap around” free C3b and other reports have determined this to be unlikely (29). Our results support this conclusion and suggest that the affinity of Factor H for non-surface-attached C3b is ~0.1 μM under physiological conditions of salt and pH and that this affinity is primarily due to the interaction between the N-terminal site and C3b.

### Measurement of the Specificity of Factor H Sites for Different C3 Fragments

**Figure 6:**
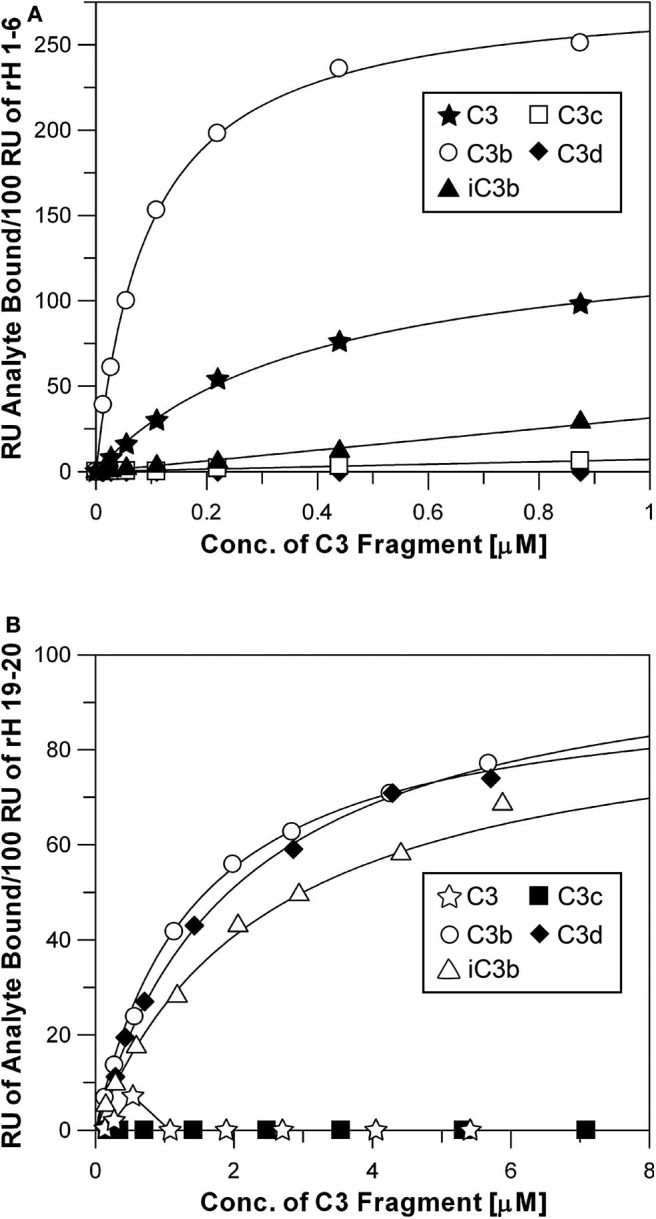
Specificity of rH 1-6 and rH 19-20 for C3 and degradation products of C3. **(A)** Binding curves of rH 1-6 with different C3 fragments. The assays were performed in the same way as described in Figures 2, 3. Each data point was generated from at least three separate binding assays. Different concentrations (0.01–0.88 μM) of native C3, C3b, iC3b, C3c, and C3d in HBS-P were injected over the chips as described in Figure 3. The *K*_*d*_ was determined by fitting the data by non-linear regression using the GraFit 5 program as described in Figure 4. **(B)** Same as above, except that purified rH 19-20 was attached to the NTA-Ni surface and higher concentrations (0.1–7.1 μM) of the C3 fragments were used in order to approach saturation with this lower affinity site.

The N-terminal and C-terminal sites of Factor H exhibit very different specificities for different C3 metabolic fragments. Figure 6 shows saturation binding curves of data generated as shown in Figure 3. Increasing concentrations of C3, C3b, iC3b, C3c, and C3d were injected over sensor chips bearing rH 1-6. The binding data was fit by nonlinear regression analysis to a one-site binding equation as described for Figure 4 to determine the K_d_ for each C3 fragment (Table 2). The N-terminal site which bears all of the complement regulatory functions of Factor H showed (Figure 6A) a significant affinity for native C3 (0.4 μM) and only a 5-fold stronger affinity for C3b (0.08 μM). In order to eliminate the concern that this might be due to C3(H_2_O) in C3, the native C3 was separated from traces of C3(H_2_O) by chromatography on Mono S (33) just prior to SPR analysis. No reduction in binding was observed with re-purified C3. Furthermore, as shown in Figure 6B this C3 did not exhibit binding to the rH 19-20 binding site providing additional assurance that the C3b-like C3(H_2_O) was not responsible for this observation. It is interesting to note that the capacity of rH 1-6 for native C3 (155 ± 8 RU) was almost exactly half that observed for C3b binding (296 ± 4 RU). The data predict that at the plasma concentration of C3 (6.5 μM) much of the Factor H in plasma (2.6 μM) will be associated with C3. Conversion of C3 to C3b resulted in a 5-fold increase in affinity for the N-terminal rH 1-6 site. The interaction with C3b was the strongest affinity observed between any C3 fragment and any site on Factor H. Inactivation of C3b by conversion to iC3b was accompanied by a 63-fold drop in the affinity for the rH 1-6 site (Table 2). No interaction between rH 1-6 and the breakdown products of iC3b (C3c and C3d) was observed (Figure 6A and Table 2).

**Table 2:** Specificity and affinity^a^ of the N- and C-terminal regions of Factor H for different C3 fragments.

**C3 fragment**	***K**_**d**_* **(μM)**^**b**^	
	**Factor H domains 1–6**	**Factor H domain 19-20**
C3	0.4 ± 0.05	NDB^c^
C3b	0.08 ± 0.04	1.7 ± 0.1
iC3b	5.0 ± 2.2	2.8 ± 0.3
C3c	NDB^c^	NDB^c^
C3d	NDB^c^	1.9 ± 0.1

a *Binding data was generated as in Figure 3 and K_d_ values were determined as in Figure 4 for the recombinantly generated Factor H fragments indicated*.

b *Standard errors from non-linear regression analysis are reported*.

c *NDB indicates “No Detectable Binding” was observed*.

The C-terminal binding site composed of CCP domains 19-20 exhibited no detectable affinity for native C3 or for the C3c fragment. However, this site had very similar affinities for C3b, iC3b, and C3d (Figure 6B and Table 2). The proposed role of this site is to hold Factor H on host cell surfaces so that the N-terminal site can regulate complement activation (1, 5, 36, 42). Thus, it would be advantageous for this site to bind equally to all three forms of C3 that remain covalently attached to the surface.

### Measurement of Heparin-Binding by Factor H Domains

**Figure 7:**
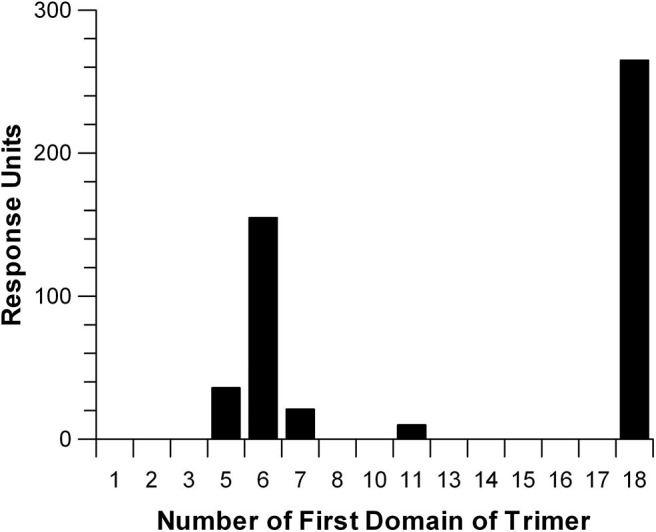
Relative affinity of three-domain Factor H fragments for heparin. Recombinant three-domain fragments of Factor H were injected over a sensor chip coated with heparin which was attached through a single amino group incorporated at the reducing end of the polysaccharide chain. This yields heparin with a single orientation and with all sulfate groups intact. The SPR measurements shown are the result of injecting each fragment at a concentration of 3 μM in HBS-P over the heparin-coated surface. Individual sensorgrams for each three-domain fragment are shown in Supplemental Figure 1.

In an effort to express a family of three domain fragments spanning the entire length of Factor H, PCR was performed to create eighteen clones each containing three domains and each shifted by one domain covering the entire length of Factor H (Figure 1). These recombinant Factor H domains were expressed in Pichia pastoris using the modified pPICZα vector as described in Experimental Procedures. As shown in Figure 1, three proteins were not expressed well while most gave very high yields. Nevertheless, because of the two domain overlap all regions of the molecule were represented by at least two different expressed proteins. Following purification, many of these proteins were found to contain a high content of carbohydrate attached by the yeast cells. Treatment with recombinant Endo H was found to remove this carbohydrate and resulted in proteins of the expected molecular weights. This family of proteins was injected one at a time at 3 μM concentration over a heparin-coated CM5 BIAcore chip (GE Bioscience) in HBS-P buffer. The method used to couple heparin (38) may account for the differences in binding observed in this study compared to other studies (6, 19, 30). Most heparin-coupled columns and chips use partially desulfated heparin (38). The desulfation procedure exposes amino groups that are used for the coupling reaction, but partially desulfated heparin exhibits different protein binding properties compared to native heparin (30). In this study a single new amino group was chemically added at the reducing end of each heparin chain (38) without removal of sulfate groups. The results of heparin affinity measurements are shown in Figure 7. The sensorgrams for each of these binding assays are presented in Supplemental Figure 1. There were three recombinant proteins representing three regions of Factor H that bound to heparin (rH 6-8, rH 11-13, and rH 18-20). The affinity of each of these for heparin was determined by injecting a series of concentrations of the recombinant protein over the heparin-coated chip. The equilibrium binding levels, measured as in Figure 3 for C3b binding, were then plotted against the concentration of ligand (Supplemental Figure 2) to determine the affinity of each region for heparin. It should be pointed out that the affinity determined for CCP 11-13 and CCP 18-20 for heparin were only estimates due to the fact that the highest free analyte concentrations attainable did not reach the K_d_ value. Table 3 lists the estimated affinity of the three sites in Factor H for heparin as well as the apparent affinity, measured in the same way on the same chip, of native full-length Factor H for heparin. As shown in Table 3, the rH 6-8 protein encompassing the known polyanion binding site in domain 7 exhibited the strongest affinity for native heparin (K_d_ = 1.2 + 0.08 μM) which was only slightly weaker than the apparent affinity of full-length Factor H (K_d_ = 0.49 + 0.08 μM). The second strongest binding site, the CCP 19-20 site at the C-terminal of Factor H, exhibited a K_d_ estimated to be 4.9 + 0.7 μM which was 10-fold weaker than that for full-length Factor H (Table 3). The weakest affinity was observed for the site in the rH 11-13 fragment (K_d_ = 17 + 3.3 μM). The existence of a polyanion binding site at this location is controversial. This site has been observed to be a polyanion binding site in a number of previous studies (16, 18, 19) while other studies have failed to find any binding in this region of the protein (13, 19). However, no previous study has quantitated the relative affinity of all three sites and most of these studies used partially desulfated heparin coupled to agarose, to biotin, or to other surfaces (6, 19, 29, 30, 43). Thus, it is reasonable to suggest that the affinity measured here might have been undetectable in some of these studies. Our evidence agrees with those reports suggesting the existence of three separate polyanion binding sites in Factor H. Furthermore, these results are in agreement with reports locating the third site between domains 12 and 15 (16, 18, 19, 40).

**Table 3:** Affinity^a^ of Factor H polyanion binding sites^b^ for heparin.

**Factor H domains**	***K_***d***_* (μM)^**c**^**
CCP 1-20	0.49 ± 0.08
CCP 6-8	1.2 ± 0.08
CCP 11-13	17 ± 3.3
CCP 18-20	4.9 ± 0.7

a *Binding regions were identified as described in Figure 7 and K_d_ values were determined as described in Supplementary Figure 2 for full-length Factor H and the trimeric recombinant proteins*.

b *Three domain recombinant proteins were expressed in yeast and purified as described in Experimental Procedures*.

c *Standard errors from non-linear regression analysis are reported*.

## Discussion

**Figure 8:**
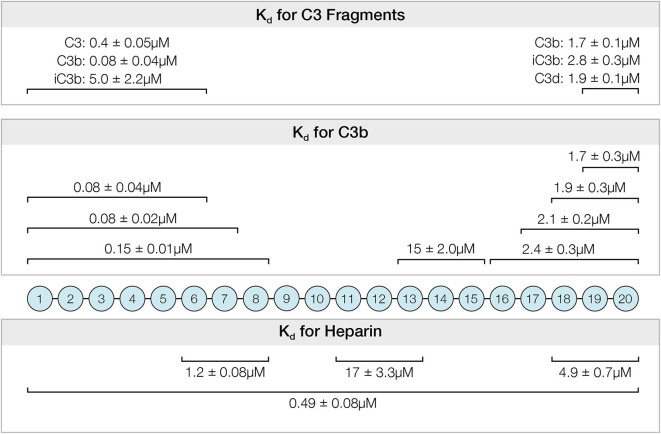
Summary of affinities determined for the interactions between recombinantly expressed fragments of Factor H and C3, C3-derived proteins and heparin. The *K*_*d*_ and standard errors for the interactions are given above the CCP domains of indicated fragments.

Activation of the complement alternative pathway is spontaneous and continuous. It must be inhibited or severe tissue damage will result as illustrated in diseases such as PNH, aHUS, and MPGN. Factor H is the primary regulator of this system and its interactions with host polyanions and with C3b and the breakdown products of C3b are key interactions controlling alternative pathway activation. Although many studies have examined individual sites of Factor H, this study presents a quantitative comparison under similar conditions of the affinities and specificities of the six known binding sites located among the twenty domains of Factor H (Figure 8). These data allow a comparison of the relative contributions of different regions to the complement regulatory functions of Factor H.

The first C3b binding site of Factor H to be localized was shown in 1984 to be in the first six and a half CCP domains at the N-terminal of Factor H (22). That study showed that both the decay accelerating and Factor I cofactor activities of Factor H were localized in this fragment. Subsequent studies demonstrated that the first four domains of Factor H were the minimal number of domains sufficient to exhibit these functions (17, 23–25, 41). Other studies (25, 27, 44) have found that additional domains contribute significantly to the activity adding an order of magnitude or more to the activity of CCP 1-4. In the work presented here we have found that the affinity of CCP 1-6 for fluid phase C3b is nearly identical to the affinity of full-length Factor H for fluid phase C3b when assayed under identical conditions. Each exhibits an affinity ~100-fold higher that others have reported for the minimal fragment CCP 1-4 (45–47). The crystal structure of C3b bound to CCP 1-4 suggests that interactions between C3b and domains CCP 5 and/or 6 could be the origin of these differences (48). Alternatively, domains 5 and 6 may stabilize the optimal conformation of CCP 1-4 for interaction with C3b.

Whereas, we have determined the *K*_*d*_ between C3b and the N-terminal domains of Factor H to be ~0.1 μM, others have found affinities of 8–15 μM (19, 27, 29, 44–48). Most of the other studies used CCP1-4 whereas our constructs included CCP domains 5 and 6. Several reports did include additional domains beyond domain 4 and found 2.5- to 100-fold greater enhancement of activity over that of the minimal fragment CCP 1-4 (25, 27, 44, 49). A report measuring binding of fluid phase C3b to surface-bound CCP fragments found full length Factor H and CCP 1-6 to have similar affinity for C3b and this affinity was ~100-fold greater than that of CCP 1-4 [Figure 4 in (25)], which quantitatively agrees with our results. Finally, our measurement of the interaction of full-length Factor H attached in the same way as our CCP 1-6 through 6His at the C-terminal showed an affinity of 0.095 μM for soluble C3b (Figure 5). This is very similar to that measured with CCP 1-6 and because this is the highest affinity site in Factor H (Figure 4 and Table 1) this interaction would be expected to dominate the binding measurements in full length Factor H. Unlike our finding of similar *K*_*d*_ of C3b for CCP 1-6 and 1-7 (i.e., 0.08 μM) (Table 2), a recent report established an affinity of 6.13 μM for CCP 1-6, while obtaining 1.03 μM for CCP 1-7 (49). In our study, the CCP 1-6- and 1-7-V5-6His protein was attached to a Ni-NTA chip, which positions the N-terminal CCP 1-5 domains pointing outward into the solution for interacting with soluble C3b, while most other studies have bound C3b to the biosensor chip surface. This, together with the different recombinant protein construct itself, may explain, at least in part, the discrepancy observed with the *K*_*d*_ of CCP 1-6 (49). Perhaps in order to rapidly inactivate fluid phase C3b during complement activation Factor H employs a high affinity interaction involving CCP 5 and/or CCP 6 which has not been seen with CCP 1-4 or surface-attached C3b used in previous studies. It is also possible that this high affinity for fluid phase C3b is critical for control of spontaneous alternative pathway activation in blood.

While it is well-established (50) that iC3b does not bind Factor H as well as C3b, we have demonstrated that the affinity is 63-fold weaker, but still significant at 5.0 μM. This may be important due to the fact that on a surface activating complement most of the C3b will be converted to iC3b and holding Factor H near that surface through multiple sites may be important for limiting excessive activation. No affinity was detectable between CCP 1-6 and C3c or C3d. However, native C3 was found (Figure 6A and Table 2) to interact with this site with an affinity only 5-fold weaker (0.4 μM) than with C3b. This unanticipated finding was rigorously examined and could not be attributed to contaminating C3b-like C3 (C3H_2_O) in the C3 preparation. In addition, this C3 showed no affinity for the C-terminal site (CCP 19-20) which exhibits easily detectable affinity for all forms of C3 breakdown fragments except C3c thus confirming that no C3b-like material contaminated the C3 preparation used. In blood, with C3 at 6.5 μM and Factor H at 2.6 μM, a *K*_*d*_ of 0.4 μM would suggest that most of the Factor H circulates in association with native C3. This interaction may be critical for controlling spontaneous alternative pathway activation in blood. It is important to note, however, that as shown in Figure 6A and described in Results, the capacity of rH 1-6 on the SPR chip for C3b was 296 ± 4 RU while the capacity for native C3 was half that at 155 ± 8 RU possibly suggesting a different mode of binding for C3 compared to C3b. If the 2-fold capacity difference was due to binding of one C3 by two rH 1-6 molecules on the SPR chip surface then the association of free C3 and Factor H in blood would be expected to be considerably reduced.

An additional consideration suggests that the mode of binding between native C3 and rH 1-6 may be different from that with C3b. The crystal structure of the complex between rH 1-4 and C3b (48) revealed that all four Factor H domains interacted with sites spanning 100 Å across the surface of C3b. In native C3, three of these four binding sites are inaccessible. Lost in C3 would be the contacts between CCP 1 and α'-NT and MG7, the contacts between CCP 3 and MG2 and CUB, and the contacts between CCP4 and the MG1 and TED domains of C3b. Only the binding of CCP 2 to MG6 has the possibility to be available in C3 and this site alone is unlikely to support an affinity only 5-fold lower that with C3b. This supports the suggestion from Figure 6A that the sites of interaction between native C3 and the N-terminal of Factor H are different from those of C3b.

While the focus of this paper is the elucidation of the relative functions of the independent sites on Factor H, clearly when assembled into intact Factor H these sites will exhibit either positive or negative cooperativity. One possible source of such effects is the folded back structure full length Factor H may assume in solution (51–53). It is not yet clear whether the N-terminal or the C-terminal folds back so speculating on the functional effect of this folding is not useful. However, what is clear is that the understanding of synergistic or antagonistic effects will be aided by the quantitative analysis of the properties of each isolated site presented here.

The existence of a C3b binding site in the center of Factor H has been difficult to confirm due to its weak affinity for C3b. It was first proposed to exist from a functional examination of deletion mutants of Factor H expressed without the central domains CCP 6-10 or 11-15 (17). Three subsequent studies seemed to confirm the existence of a binding site in the central region by using antibodies or by examining effects on cofactor activity, decay acceleration function, and target recognition (39–41). A later study also found evidence of a C3b binding site in the center domains of Factor H, but concluded that binding was so weak that it was insignificant compared to that at the N-terminal and C-terminal sites (19). The preponderance of the published evidence together with the results presented here in Figure 4C and Table 1 seem to support the existence of a specific C3b binding site in the center of Factor H. In addition, all of the studies agree with our results showing that the affinity is low compared to the other two C3b binding sites located at the ends of the protein. The weak affinity of the central site, we estimate to be 100-fold weaker (Table 1) relative to the N-terminal site, may be the reason that some studies failed to observe binding to C3b. Nevertheless, as correctly stated by others (19) a contribution to cooperative binding with the other sites cannot be ruled out and as such it could have a significant effect on Factor H function far beyond its apparent low affinity when measured in isolation. The functional studies cited above support a role for this site in the Factor I cofactor, decay acceleration and perhaps host recognition functions of Factor H (39, 41).

The exact location of the central C3b binding site is controversial. The original identification of this site relied on binding changes resulting from deletion of CCP 6-10 or 11-15 (17). The results with CCP 6-10 deletion may have been due to the loss of the polyanion binding site in CCP 7. Evidence presented in Table 1 shows weak binding (15 ± 2 μM) of C3b to rH 6-15, but no binding with rH 6-12 suggesting that the binding site localizes to CCP 13-15. The region at or near CCP 12-13 is supported by much of the current experimental evidence, although Schmidt et al. (19) found weak binding between C3b and CCP 6-8 and 7-8 constructs. Although, this paper (19) also reported binding with a CCP 8-15 construct, little or no binding was observed with CCP 8-9, 10-15, 10-12, 11-14, 12-13, or 13-15. Jokiranta et al. failed to detect C3b binding to CCP 8-11 or 15-18 while showing C3b and C3c binding to CCP 8-20 in line with the C3b/C3c site being located near CCP 12-14 (41). In support of this conclusion, a peptide from CCP 13 was shown to enhance complement-mediated lysis of human cells, to compete with Factor H binding to cells and to decrease the cofactor activity of Factor H on C3b (54). In contrast with this conclusion, Schmidt et al. saw no effect of purified CCP 12-13 on complement activation (44). As mentioned above negative binding results may have been due to experimental conditions that did not favor detection of low affinity binding. Thus, far no disease association to this site has been found, but if this site has functional importance then genetic studies should eventually allow us to identify its true location, its biological function, and its importance to the regulation of complement.

While not having any direct complement regulatory activity, the C-terminal of Factor H determines whether the alternative pathway activates or not on a given target. The presence of sialic acids or other polyanions on a surface increases the affinity between C3b and Factor H ~5- to 10-fold (2, 3) and recognition of polyanions was shown to be through Factor H, not C3b (4). The C-terminal 19-20 domains alone were shown to exhibit a 5- to 10-fold greater affinity for C3b on host-like cells with surface polyanions compared to activators lacking polyanions (5) thus accounting for target discrimination by the alternative pathway (55). Mutations in the C-terminal domains that reduce the affinity for either C3b or polyanions result in reduced regulation of alternative pathway activation on host tissues and lead to complement-mediated pathology in diseases such as atypical HUS and membranoproliferative glomerulonephritis (1).

There is general agreement in the literature regarding the affinity and specificity of the C-terminal CCP 19-20 site for C3b and its products. As shown in Figure 6B and summarized in Table 1 we found the affinity (*K*_*d*_) for C3b, iC3b, and C3d to be 1.7, 2.8 and 1.9 μM, respectively. The presence or absence of CCP domains 16-18 did not significantly affect the affinity (Table 1). Published dissociation constants of this region for C3b range from 0.5 to 9 μM if one considers only studies done under conditions close to physiological pH and ionic strength (15, 19, 28, 44, 56–59). The affinity between C3b/iC3b/C3d and CCP 19-20 goes up as much as 10-fold when the ligand C3b/iC3b/C3d is attached to a surface that can also engage the polyanion binding site in CCP 19-20 (14). For example, binding of CCP 19-20 to EsC3b cells exhibits an affinity of 0.1 μM and the inhibition by CCP 19-20 of the binding of full-length Factor H to EsC3b cells showed an IC50 of 0.2 to 0.3 μM (14, 57). CCP 19-20 bound to tumor necrosis factor-α-activated mouse endothelial cells with a *K*_*d*_ of 0.1 μM (26). Inhibition of the binding of radiolabeled CCP 19-20 to EsC3b cells by unlabeled CCP 19-20 exhibited an IC50 of 0.04 μM (57). Similarly, the inhibition of hemolysis of host erythrocytes (human) has been shown to be blocked by the presence of CCP 19-20 with an EC50 of 0.4–1 μM due to the inhibition of Factor H regulation (14, 57). One additional effect of CCP 19-20 may be that this region appears to be the site responsible for the dimerization and tetramerization of Factor H (30, 36, 60–62). Thus, soluble CCP 19-20 would not only be able to block the C3b-polyanion sites on cells, but also inhibit the formation of Factor H dimers and tetramers in solution. These combined effects could explain the ~10-fold difference between the *K*_*d*_ for C3b/iC3b/C3d (~2 μM) and the IC50 and EC50 values of 0.2–0.4 μM of CCP 19-20 in the functional inhibition assays cited above. The effectiveness of CCP 19-20 for inhibiting normal regulation by Factor H has been used *in vivo* to examine the effects of reduced Factor H function on disease processes in models of arthritis and allergy (63, 64).

Factor H possesses two well-characterized polyanion binding sites and one binding site whose location and even existence is disputed. The multitude of C3b binding sites and polyanion binding sites are thought to operate cooperatively to differentiate between potential targets of complement or to enhance control on different host cells and tissues (40, 65). In 1996 Blackmore et al. localized a strong polyanion binding site to CCP 7 using heparin-agarose affinity chromatography (66). CCP 7 was later identified as the site of the common human variant Y402H strongly linked to age-related macular degeneration [reviewed in (67)]. In 1998 Blackmore et al. identified a second heparin binding site in domain 20 (13). Sialic acid binding was also localized to this C-terminal site and antibodies to CCP 19-20 blocked polyanion binding (42, 68, 69). Comparisons of affinity and specificity suggest that the sites differ significantly in their interactions with different polyanions (8). With regard to the affinity for heparin, other studies have measured the affinity between full-length Factor H and heparin (30). Using biotinylated small purified heparin fragments coupled to a BIAcore chip via streptavidin Khan et al. (30) measured a *K*_*d*_ = 0.5 μM. We determined an almost identical affinity for this interaction of *K*_*d*_ = 0.49 μM (Table 3 and Supplemental Figure 2). The affinity for the individual sites was measured by Khan et al. using biotinylated heparin fragments (dp32) so direct comparisons cannot be made, but the affinities they measured for full-length Factor H, CCP 6-8, and CCP 16-20 were 2.7, 4.3, and 20 μM, respectively, (30). Our measurements, shown in Table 3, with full-length Factor H, CCP 6-8, and CCP 18-20 showed binding constants for heparin of 0.49, 1.2, and 4.9 μM, respectively. The ratios of affinities from the two studies agree well at 1/1.6/7.4 (30) vs. 1/2.4/10 (Table 3 and Supplemental Figure 2), respectively. Thus, both studies show that the polyanion site in domain 7 has a 4- to 5-fold higher affinity for heparin than the C-terminal site. This ratio may only hold true for heparin, however, because the sites differ in specificity (6, 8, 18, 68).

The third polyanion binding site, which numerous publications locate in the center of Factor H (16, 18, 19) exhibits an affinity for heparin only 3- to 4-fold weaker than that of CCP 19-20 (Table 3 and Supplemental Figure 2). Under physiological conditions of pH and ionic strength (HEPES-buffered saline, pH 7.4) the *K*_*d*_ for the interaction of CCP 11-13 with heparin was found to be 17 ± 3.3 μM which is only 14-fold weaker that the highest affinity site in Factor H located in CCP 7 (Table 3). Most published studies have measured the salt sensitivity of polyanion binding rather than the affinity (13, 18, 19, 43, 66). These measurements are generally made by comparing the elution position of fragments from heparin-agarose columns as they are eluted with a salt gradient. The salt sensitivity of binding may show no correlation to affinity. High affinity interactions can be insensitive to salt or extremely sensitive to salt and low affinity interactions can show similar diversity. While these studies may provide useful information, only measurements made under physiological salt conditions provide reliable information about affinity. Our measurements of affinity suggest that the CCP 11-13 site could be a factor in polyanion recognition, i.e., host cell and tissue recognition, especially if heparin is not the preferred polyanion for this site. The data also suggests that CCP 13 may require CCP 12 for this site to express affinity for heparin (Figure 7 and Supplemental Figure 1). In addition, CCP 12 and CCP 13 display unusual structures compared to other CCP domains (52, 65), they form a tight inflexible interface, and CCP 13 is one of the most electrostatically positive domains in Factor H (70). None of these features are likely to be present without a functional purpose. Thus, affinity measurements, functional measurements (40) and structural considerations all support the conclusion that there is a third polyanion binding site located in the center of the protein.

## Data Availability Statement

All datasets generated for this study are included in the article/Supplementary Material.

## Author Contributions

AH and MS cloned, expressed, purified recombinant proteins, and performed BIAcore binding studies. CC cloned, expressed, purified the three-domain fragments of Factor H, and performed BIAcore binding studies. MA and VF purified proteins and performed BIAcore binding studies. MP conceived the project, designed experiments, analyzed results, and wrote the paper. All authors analyzed the results and approved the final version of the manuscript.

## Conflict of Interest

During the course of this work, MP was an officer of, and had a financial interest in Complement Technology, Inc., a supplier of complement reagents. These relationships no longer exist. The remaining authors declare that the research was conducted in the absence of any commercial or financial relationships that could be construed as a potential conflict of interest.

## Abbreviations

C3b, iC3b: C3c and C3d are proteolytically generated fragments of human complement protein C3
VBS: Veronal-buffered saline
GVB: VBS containing 0.1% gelatin
HBS-P: HEPES-buffered saline containing P20 surfactant and azide
CCP: complement control protein domain also referred to as SCR (short consensus repeat) domains in the literature
ARMD: age-related macular degeneration
aHUS: atypical hemolytic uremic syndrome
MPGN: membranoproliferative glomerulonephritis
CRP: C-reactive protein
PNH: paroxysmal nocturnal hemoglobinuria.
