# Supplementary material for: Characterization of Binding Properties of Individual Functional Sites of Human Complement Factor H
Source: Front Immunol. 2020 Aug 4;11:1728. doi: 10.3389/fimmu.2020.01728 (PMC7417313; doi:10.3389/fimmu.2020.01728)
Supplement: Supplementary file 1 [file Data_Sheet_1.pdf]

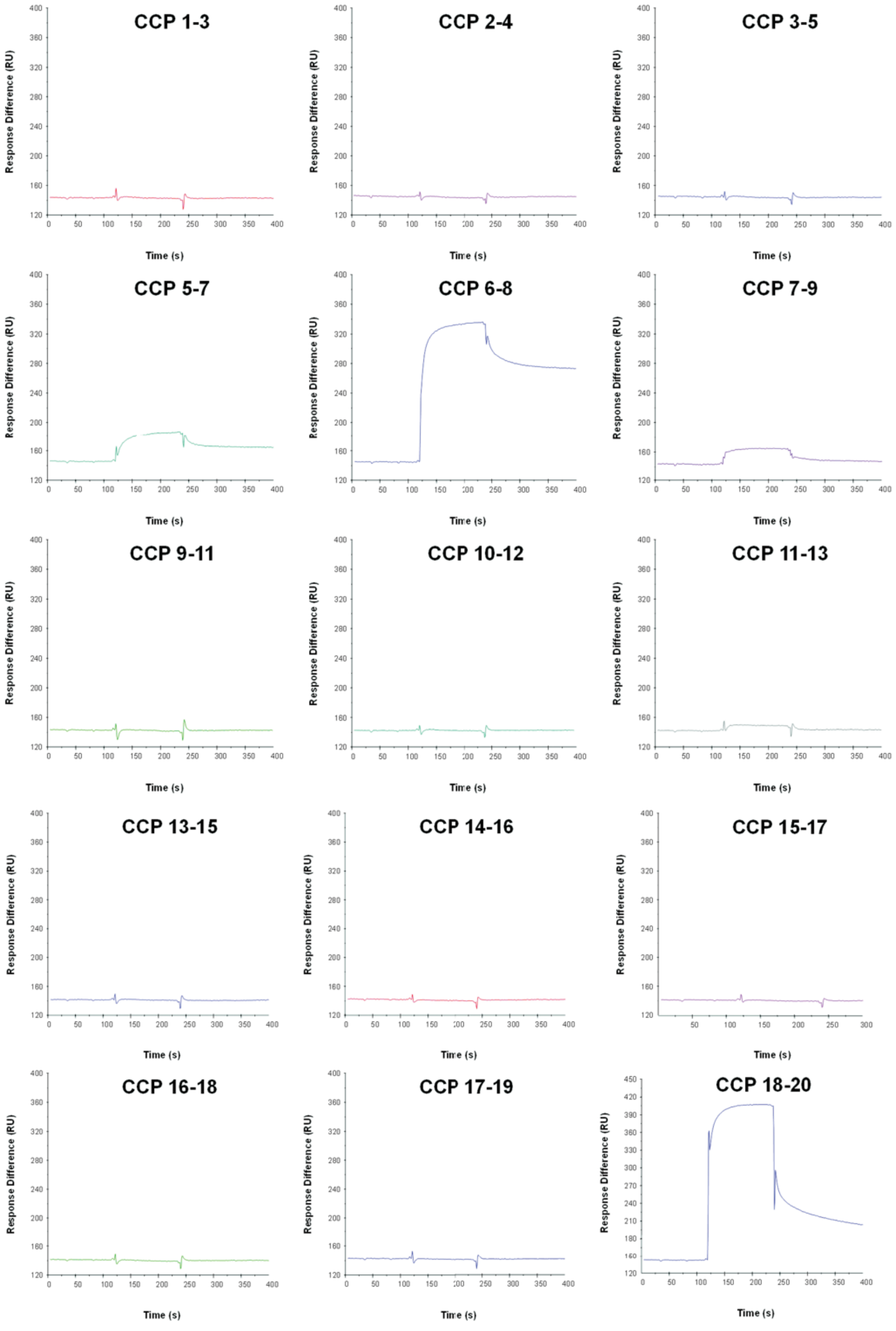

Supplementary Figure 1. Individual sensorgrams showing the binding of three domain proteins spanning the entire length of Factor H. Proteins were expressed in *Pichia*, purified, treated with Endo H to remove excess carbohydrate, dialyzed into HBS-P buffer and injected at 3 µM. The sensor surface was CM5 to which heparin had been coupled using a single amino group added to each heparin molecule at the reducing end of the glycosaminoglycan chain.

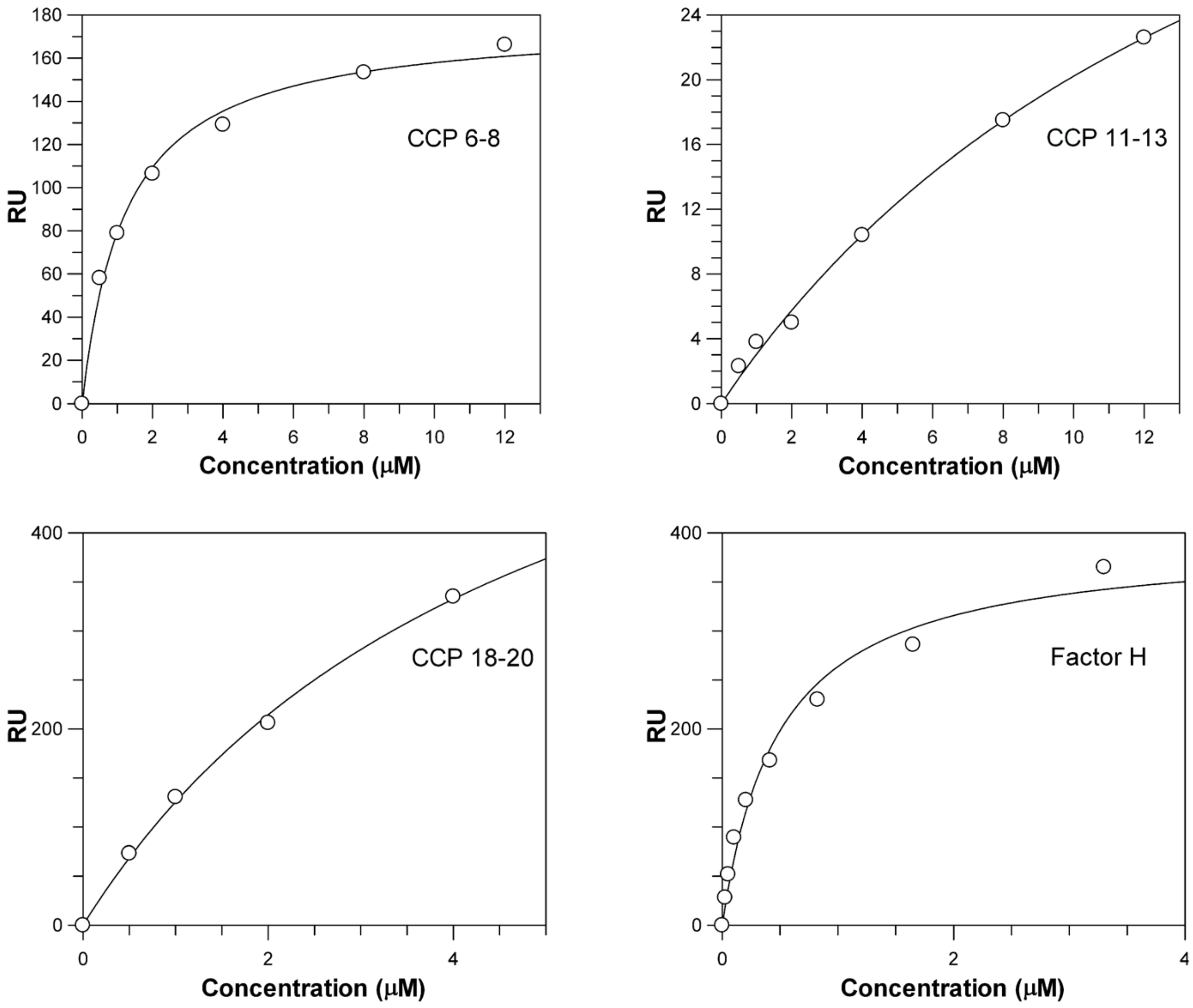

Supplementary Figure 2. Individual binding curves showing the binding of three domain proteins CCP 6-8, CCP 11-13, CCP 18-20 and full-length Factor H to the heparin-coated sensor surface. Recombinant proteins were expressed in Pichia, purified, treated with Endo H to remove excess carbohydrate, dialyzed into HBS-P buffer and injected at the indicated concentrations. Full-length Factor H was purified from human plasma. The sensor surface was CM5 to which heparin had been coupled using a single amino group added to each heparin molecule at the reducing end of the glycosaminoglycan chain. Each data point represents the equilibrium saturation level from one individual ligand binding curve from experiments like those described in Supplementary Fig. 1. The *Kd* (shown in Table 3) was determined by fitting the data using nonlinear regression to a single site ligand binding equation (Bound = (Capacity*[Free])/(*Kd*+[Free])) using GraFit 5 program (Erithacus). Note that the fit to a single site binding equation for the Factor H binding to heparin is not ideal as would be expected for a molecule with multiple binding sites. This single site model was applied in order to obtain a weighted average affinity of the different contact regions for heparin in the Factor H molecule.
